# Mechanical Properties of 3D-Printing Polylactic Acid Parts subjected to Bending Stress and Fatigue Testing

**DOI:** 10.3390/ma12233859

**Published:** 2019-11-22

**Authors:** J. Antonio Travieso-Rodriguez, Ramon Jerez-Mesa, Jordi Llumà, Oriol Traver-Ramos, Giovanni Gomez-Gras, Joan Josep Roa Rovira

**Affiliations:** 1 Mechanical Engineering Department, Escola d’Enginyeria de Barcelona Est, Universitat Politècnica de Catalunya, Avinguda d’Eduard Maristany, 10–14, 08019 Barcelona, Spain; oriol.traver@upc.edu; 2 Engineering Department, Faculty of Sciences and Technology, Universitat de Vic—Universitat Central de Catalunya, C. Laura, 13 Vic, 08500 Barcelona, Spain; ramon.jerez@uvic.cat; 3 Materials Science and Metallurgical Engineering Department, Universitat Politècnica de Catalunya, Escola d’Enginyeria de Barcelona Est, Avinguda d’Eduard Maristany, 10–14, 08019 Barcelona, Spain; jordi.lluma@upc.edu (J.L.); joan.josep.roa@upc.edu (J.J.R.R.); 4 Industrial Engineering Department, IQS School of Engineering, Universitat Ramon Llull, Via Augusta, 390, 08017 Barcelona, Spain; giovanni.gomez@iqs.url.edu

**Keywords:** additive manufacturing, 3D printing, fused filament fabrication, flexural properties, fatigue, PLA

## Abstract

This paper aims to analyse the mechanical properties response of polylactic acid (PLA) parts manufactured through fused filament fabrication. The influence of six manufacturing factors (layer height, filament width, fill density, layer orientation, printing velocity, and infill pattern) on the flexural resistance of PLA specimens is studied through an L27 Taguchi experimental array. Different geometries were tested on a four-point bending machine and on a rotating bending machine. From the first experimental phase, an optimal set of parameters deriving in the highest flexural resistance was determined. The results show that layer orientation is the most influential parameter, followed by layer height, filament width, and printing velocity, whereas the fill density and infill pattern show no significant influence. Finally, the fatigue fracture behaviour is evaluated and compared with that of previous studies’ results, in order to present a comprehensive study of the mechanical properties of the material under different kind of solicitations.

## 1. Introduction

Manufacturing through fused filament fabrication (FFF) or 3D-printing is a phenomenon that has drastically changed the way manufacturing is understood, mainly during the last decade [1]. The interest comes from the clear advantages that this group of technologies presents with respect to traditional manufacturing technologies; that is, great freedom of design and innovation capacities, a stronger connection between design and manufacturing, or the ability to manufacture unique pieces [2]. In addition, additive manufacturing (AM) systems have been easily implemented in domestic or low-scale manufacturing environments as a cheap and easy manufacturing technology.

Regardless of the rapid expansion of AM, the problem related to the identification and prediction of the mechanical behaviour and physical characteristics of the final pieces has been the main handicap for its application in industrial environments or final pieces. This difficulty lies in the fact that the parameters to be defined during the manufacturing process are numerous and interact with one another; and, on the other hand, because of the anisotropy of the material, caused by the high influence of the filament orientations in the manufacturing space [3]. Furthermore, anisotropy also originated thanks to the difference between the bonding forces between strands of the same layer (intralayer) and between layers (interlayer) [4]. For these reasons, the orientation of the layers is a key parameter to be defined when taking into account the work conditions of the piece.

According to Bellehumeur et al. [5], the mechanical resistance of parts is the result of the addition of three factors: the resistance of the filaments, the resistance of the union between filaments of the same layer, and the resistance of the union between layers. The inherent resistance of the filaments mainly depends on the mechanical properties of the raw material and the strength of the joints depends on the cohesion between filaments. This is proportional to the thermal energy of the filaments when they come into contact when being placed. The union is a local sinter in which polymer chains are shared. This process is applicable to all joints, between layer threads of both the same layer and different ones.

The authors Gurrala and Regalla [6], Gray et al. [7], and Zhong et al. [8] agree that the orientation of the layers must be coincident with the directions of the expected service loads to optimize the mechanical properties. In contrast, in compression forces, owing to the buckling effect, the fibres tend to bend. Therefore, the fibres should be oriented perpendicular to the load in this case [9].

This same effect of the orientation of the layers on the mechanical properties of the workpieces, has also been observed in other processes of AM, as in the technology of laminated object manufacturing (LOM), according to Olivier et al. [10]; selective sintering by laser, as reported Ajoku et al. [11]; or stereolithography presented by Quintana et al. [12].

Another parameter with great influence on the mechanical properties is the height of the layer. When the layers have a lower height, the parts show an overall better cohesion between layers, because the contact surface is greater and the empty space between filaments is smaller. This effect improves the transport mechanism of thermal energy, favouring the welding between wires, as found in the work of [9]

On the other hand, the thickness or width of the extruded filament is also a parameter that significantly influences the mechanical behaviour. It has a great impact on the transport mechanisms of thermal energy, which will affect the cohesion of the threads, according to the study proposed by Wang et al. [13].

**Figure 1:**
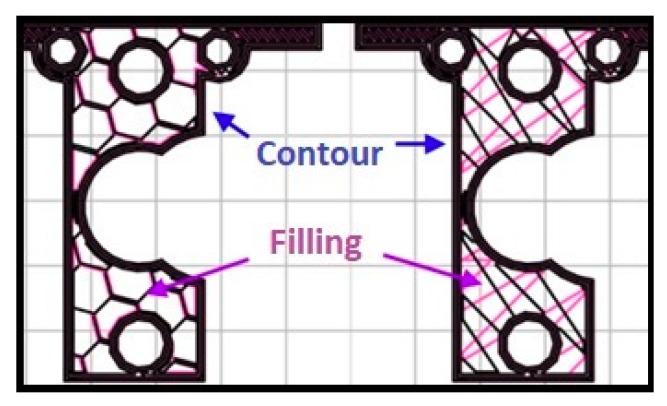
Section of a piece printed with two types of fill patterns. Left: honeycomb, right: linear.

The printing strategy determines the paths of the machine head in the creation of the piece. Within this context, the printed pieces are composed of two characteristic zones: the contour and infill. The outline is the skin that delimits the piece and corresponds to the outer perimeters. The infill is the one formed by the trajectories that the nozzle follows to fill the empty space that remains inside the contour, as depicted in Figure 1.

Generally, in each layer, the contour is first performed followed by the internal filling with the selected printing strategy. Each one provides different mechanical properties. In the present work, the influence of several patterns shall be studied, as well as different infill densities, to assess their impact on the workpiece flexural behaviour.

The printing velocity is also a modifiable parameter. It can be defined for each printing zone, being independent for the contours, fills, and upper and lower layers. The velocity will be a parameter of study in this work since it has influence in the process of melting and solidification of the filaments. In addition, it affects the rate of extruded material.

Considering the aforementioned base of knowledge about FFF, this paper aims to study the influence of the manufacturing parameters on the mechanical properties of pieces made of polylactic acid (PLA) manufactured by FFF. Specifically, the flexural mechanical properties of these parts are evaluated. The results obtained are also compared with the those obtained in a previous study by Gómez-Gras et al. [14] and Jerez-Mesa et al. [15], performed on the same material subjected to a different loading mode. The main novelty delivered by this paper is that it contributes to the enrichment of mechanical behavioural data regarding PLA material. So far, an extensive study about bending properties and their direct comparison to fatigue performance linked to process parameters has not been found in the literature. For this reason, the results presented in this paper complement other results regarding tensile or fatigue properties, presented by authors in previous references, as presented above. The makers and users of FFF machines often ask about the best way to manufacture their parts. The answer should be that printing parameters should be chosen according to the expected part behaviour; this paper contributes to enriching that answer.

## 2. Materials and Methods

In this paper, the flexural mechanical properties of PLA are assessed. The influence of the manufacturing parameters in these properties will also be analysed. Therefore, the first experimental stage explained in this paper comprises a series of four-point bending tests performed on prismatic test specimens, following the American Society for Testing and Materials (ASTM) D6272-2 standard [16].

To better understand the influence of the significant parameters, different images of the fractured areas were taken and subsequently analysed. In addition, to complement the fractography, a micro scratch test was performed, which helped to better understand the fracture mechanism of the pieces.

In a second experimental stage, a fatigue Whöler curve generated through flexural fatigue tests was drawn to analyse whether the best conditions obtained in the four-point bending tests also derive in good fatigue properties.

### 2.1. Four-Point Bending Tests

#### 2.1.1. Specimens Manufacture

**Figure 2:**
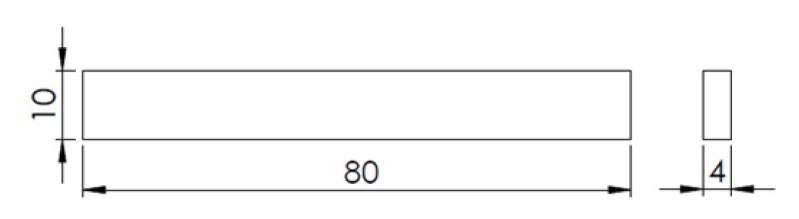
Test specimen’s geometry: 80 mm × 10 mm × 4 mm, according to the D6272-02 ASTM standard.

The design of the specimens used in the study was done with SOLIDWORKS^®^ Research Edition 2019 software (Dassault Systèmes, Vélizy-Villacoublay, France) and the models were filleted with Slic3r software (GNU Affero General Public License) [17]. Subsequently, they were manufactured in the domestic 3D printer, Pyramid 3Dstudio XL Single Extruder. Their geometry is shown in Figure 2, with dimensions according to the standard that governs the bending test. All manufactured specimens were submitted to a quality control, in which they were weighed and measured with a calliper. Therefore, they had to be validated before testing from a dimensional and constructive point of view. The resulting lengths, widths, and weights were statistically processed, and those specimens whose descriptors were out of the ±2% were considered not to comply and were immediately discarded.

The material used in the manufacture of the specimens, as discussed above, is PLA. It is a biodegradable thermoplastic. The choice of PLA as the study material was based on the fact that it is the most used material in domestic 3D printing. In this case, the selected filament was manufactured by Fillamentum Company from the Czech Republic. It has a diameter of 3 mm and its extrusion temperature is around 210 °C. The technical information provided by the manufacturer is indicated in Table 1.

#### 2.1.2. Taguchi Experimental Design

To carry out the four-point bending study, the design of experiments (DOE) technique was used. The design consists of the combination of the printing parameters that are considered most influential in mechanical behaviour. Six parameters are included in the study, and three levels of each one are defined (Table 2). They were selected taking into account the bibliography studied, as well as the experience of previous work of the research group.

**Figure 3:**
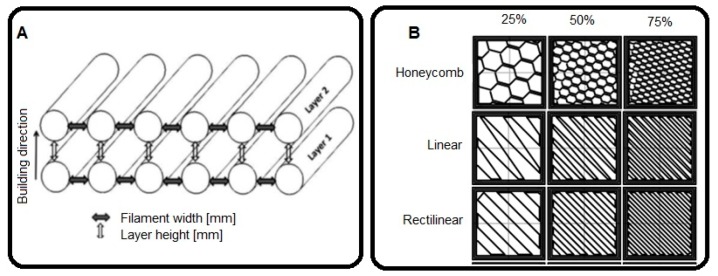
Schematic representation of the parameters used in the study: (**A**) filament width and layer height, (**B**) infill pattern and fill density.

Filament width: Determined by the diameters of the extrusion nozzles: 0.3, 0.4, and 0.6 mm. It defines the volume and surface of the extruded threads, as well as the welding surface between filaments (Figure 3A).

Layer height: Describes the thickness of each layer and, therefore, the number of layers the printed piece will have. It affects the volume and surface of the threads, as well as the welding between layers. The manufacturing time is inversely proportional to the layer height. Thinner layers imply more layers to print and a longer production time (Figure 3A).

Fill density: Represents the amount of material that is deposited within the contours. It avoids relative movements between contours and gives robustness to the pieces. It also determines the distance between the inner threads and affects material consumption (Figure 3B).

Fill pattern: Defines the trajectories that the nozzle follows to fill the empty space within the contour. Each pattern will create a different interior geometry producing different mechanical behaviours (Figure 3B).

**Figure 4:**
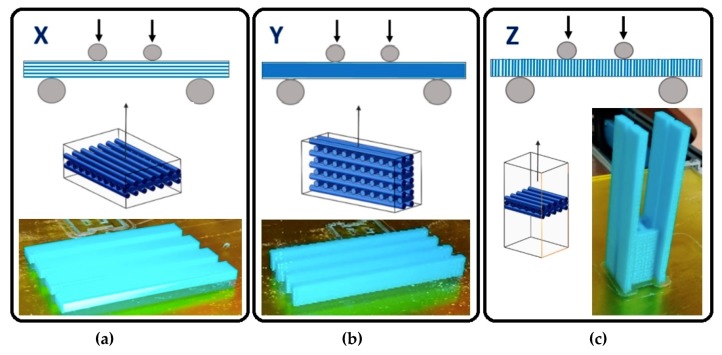
The orientation of the layers’ stacking, in the manufactured specimens. (**a**) X-axis oriented; (**b**) Y-axis oriented; (**c**) Z-axis oriented.

Orientation: The specimens will be printed in the direction of the three coordinate axes: X, Y, and Z, as shown in Figure 4. In this way, the stacking of the layers will be done in three different ways and their behaviour can be studied. Normally, the stacking direction is the most determinant factor in mechanical behaviour [18].

Printing velocity: It determines the extrusion and deposition of the threads’ velocity. The velocity is defined for each part of the piece (inner, external perimeters, inner threads, and so on) to optimize the manufacturing time. In this study, the same velocity was defined for all parts of the piece to homogenize its structure.

In this study, a Taguchi L27 DOE was used. This method has been applied successfully in other studies concerning the mechanical properties of FFF pieces [14]. Table 3 shows an orthogonal matrix with a specific combination of parameters used. The influence of these separately as well as their interaction will be studied.

The rest of the parameters that affect the conception of the test specimens remained constant.

#### 2.1.3. Experimental Setup

The tests were carried out on the Microtest EM2/20 universal electromechanical machine, with a capacity of 20 kN, displacement of 300 mm, and a speed range 0–160 mm/min. The force acquisition was performed with a load cell of 500 N and a precision of 0.03 N.

**Figure 5:**
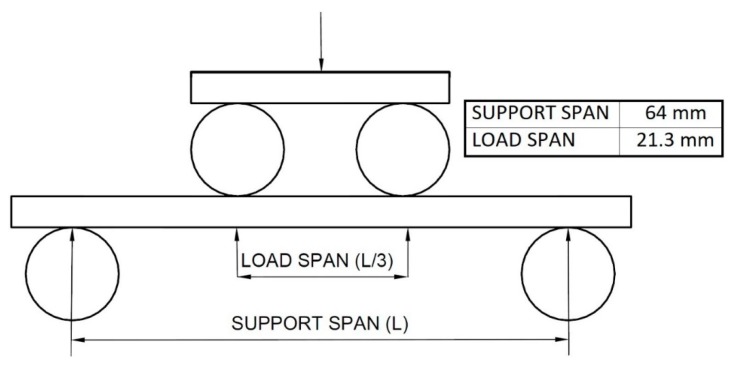
Diagram of the four-point bending test method, according to the D6272-02 ASTM standard.

The test consists of placing the specimen of a rectangular cross section over two supports and loading it at two points by means of two loading rollers; each at an equal distance from the adjacent support point. The specimen is bent at a constant speed, until the external fibres break, or until the maximum deformation of the external fibres reaches a 5% elongation. The parameters used in the experiment are described in the D6272-02 ASTM standard; that is, a support span of 64 mm and a load span of 21.3 mm (Figure 5).

**Figure 6:**
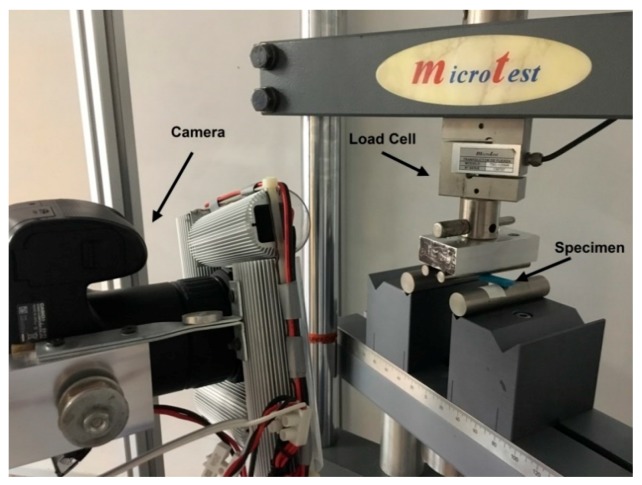
The installation used to perform the four-point bending tests.

The deflection value will be obtained through image processing. High-definition video capture is planned for all tests. That way, the displacement will be obtained through image processing, by following a marker painted on the lower fibre of the specimen. The displacement will be determined to calculate the overall deflection (Figure 6). On the other hand, the force applied by the loading rollers will be measured with a load cell. The objective of data processing is to create the stress–strain curve of the specimens [19]. From the obtained curve, the following results will be extracted: Young’s modulus (E), elastic limit (Rp_0.2_), maximum strength (σ_max_), and maximum deformation (ε).

The test method used contemplates two different types, which differ in the test speed according to the behaviour of the test piece.

Type A. Used in test specimens that break with little deflection.

Type B. Used in the test specimens that absorb large deflections during the test.

The Type A test will end when breakage is detected in the outer fibres of the test pieces, and the Type B test will end when specimens break or the deflection D *=* 10.9 mm, according to measurements of the specimens and the parameters used.

A previous experimental testing was performed to validate the adequacy of the described method. From these experiments, it was detected that specimens printed in the direction of the *Z*-axis do not admit deflection, and present brittle failure, while the specimens printed in the direction of the *X*- and *Y*-axes admit large deflections. The summary of the test types can be seen in Table 4.

#### 2.1.4. Data Analysis

**Figure 7:**
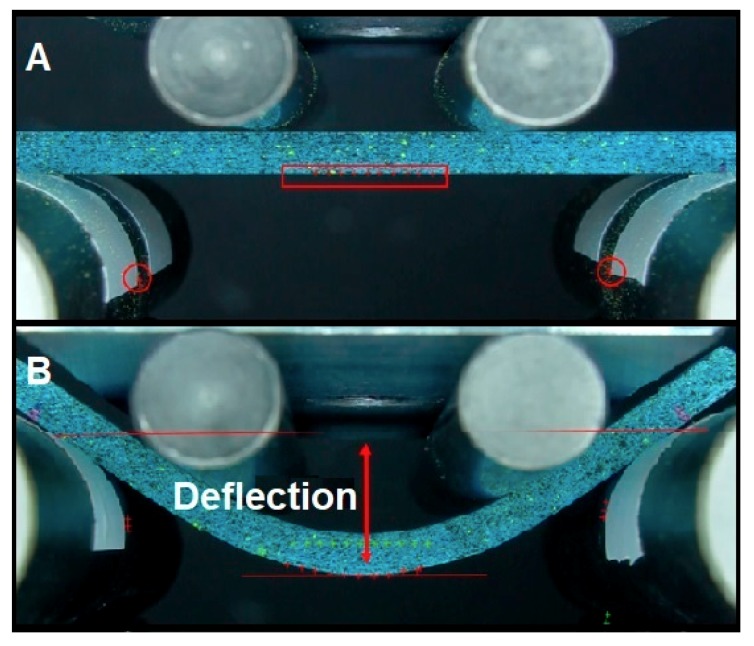
Schematic representation of the data collection process during the tests. (**A**) Initial position of markers; (**B**) final position (red crosses) and initial position (green crosses) of the markers.

The data analysis was processed by following the steps described as follows:

1. Separation of the frames of the High Definition videos of each test. The camera used registered the image at approximately 60 fps. The tests lasted between 45 s and 2 min, so, in each of the 108 tests, between 2700 and 7200 frames were processed.
2. Calculation of the specimen’s deflection through the frames. Position markers were painted on the outer fibre of the specimen, where the maximum deflection occurs, and on the static rollers (Figure 7A). The difference between the final position and the initial one, between the most displaced marker of the specimen and the markers on the static rollers, is considered the maximum deflection (Figure 7B). This analysis was performed through a self-designed MATLAB^®^ code (version 2018) with image processing functions.
  The calculation of the stress that is generated in the specimen at each moment by means of Equation (1) is as follows:
  (1) $$S=\frac{PL}{bd^{2}},$$
  where
  *S =* Stress in the outermost fibre (MPa)
  *P =* Applied load (N)
  *L =* Distance between support rollers (64 mm)
  *b =* Width of the specimen (10 mm)
  *d =* Thickness of the specimen (4 mm)
3. Analysis of the stress–strain curve obtained to extract the study parameters.

### 2.2. Fractography and Scratch Test

In order to analyse the influence of the parameters that were significant, a SMZ-168 MOTIC stereo microscope was used to observe the fractures surfaces. The most interesting fracture phenomena were photographed with a MOTICAM 2300 camera. Both equipment were manufactured by Motic^®^, Xiamen, China.

**Figure 8:**
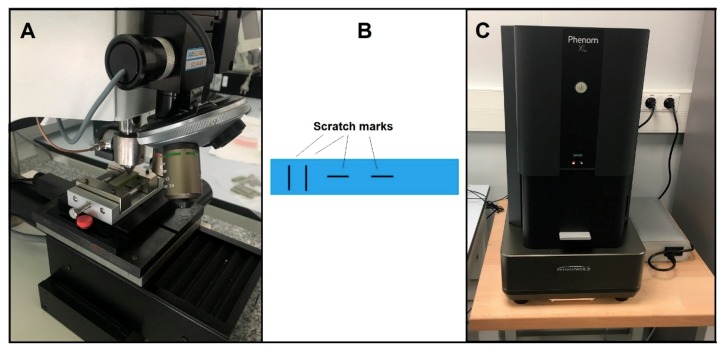
Micro scratch test. (**A**) scratch tester unit; (**B**) specimen; (**C**) scanning electron microscopy (SEM) ThermoFisher Scientific Phenom XL.

Also, micro scratch tests were conducted in a scratch tester unit (CSM-Instruments, Needham, MA, USA) (Figure 8A) using a sphere-conical diamond indenter with a radius of 200 µm. Tests were done under a linearly increasing load, from 0 to 70 N, at a loading rate of 10 mm·min^−1^ and in an interval length of 5 mm, according to the ASTM C1624-05 standard [20]. Figure 8B shows the two different scratches per specimen that were carried out in order to observe the reproducibility of the induced damage. Furthermore, the micro scratch tests were conducted in the longitudinal and transversal printing direction to observe the main plastic deformation mechanisms induced. Surface damage induced during scratch tests was observed by a desktop scanning electron microscopy (SEM) Phenom XL from ThermoFisher Scientific (Waltham, MA, USA) (Figure 8C).

### 2.3. Fatigue Test

To complete this study, it is proposed to analyse, in a second experimental stage, how cylindrical specimens behave when manufactured through the optimal parameter set found in the previous study, subjected to a rotating fatigue test. This will also allow the comparison with other values previously obtained for the same material using other printing conditions [14].

**Figure 9:**
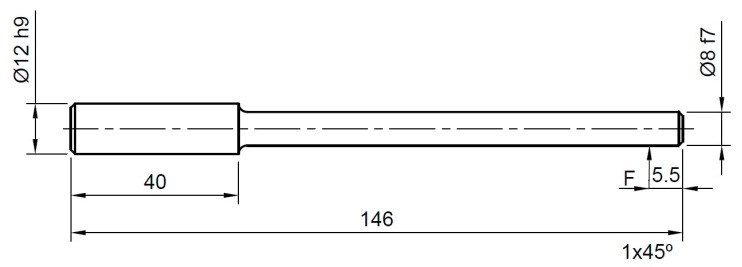
Dimensions of the test specimens used in the fatigue test.

The rotating bending fatigue test consists of applying a variable bending moment on a cylindrical test piece of known dimensions that rotates on its own axis. In this way, alternative tensile and compressive stresses are generated in the external fibres in each rotation. The test was carried out on printed cylindrical specimens like the one shown in Figure 9. For the fabrication of the fatigue specimens, the same 3D printer was used.

## 3. Results and Discussion

### 3.1. Four-Point Bending Test

Table 5 shows the results, for each printing configuration, of the stress-strain curve as the average results of the five repetitions and their standard deviation.

An analysis of variance (ANOVA) was performed on the dataset included in the Taguchi experimental array, for each parameter that describes the mechanical behaviour of the evaluated specimens. To validate the statistical relevance of the parameters included in the model, the p-value associated with the ANOVA was compared to a significance level of 5%.

One of the first observations derived from the experimental testing is that specimens printed in the *Z*-axis direction presented fragile failure, as their failure mode was governed by the lower resistance between layers deposited vertically, thus with a lower neck growth area between them. For that reason, the elastic limit (*Rp_0.2_*) associated with these specimens was by default considered equal to their maximum strength (*σ_max_*). This approach was necessary to perform the statistical analysis, and allows the brittle behaviour to be included in the statistical analysis.

Alongside the yield limit and the maximum strength, the Young’s modulus and maximum deformation were considered as response variables to analyse the influence of the different parameters in the statistical study. The following subsections describe the influence of the different parameters on the considered mechanical properties.

#### 3.1.1. Young’s Modulus

**Figure 10:**
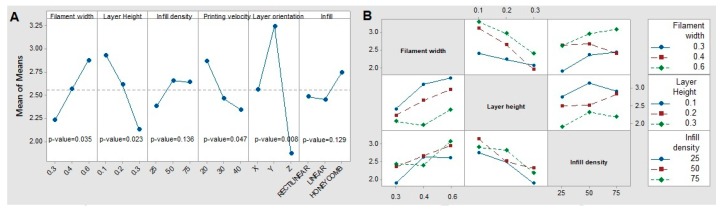
Main effects of (**A**) means and (**B**) interactions on Young’s modulus.

As a predictable result, the specimens oriented along the *Z*-axis direction present the lowest rigidity of all, owing to their described brittle behaviour, and thus can be orientation defined as the most influential parameter (Figure 10A). The highest deformation module in the elastic regime is defined by an orientation of the fibres along the *Y*-axis direction, because of the different pattern deposited in this direction with regards to the *X*--axis orientation.

On the other hand, an increase in the value of Young’s modulus occurs when the filament width increases, probably because of the higher inertia of the single filaments that restrict bending. This effect of higher inertia of the surface is also achieved by decreasing the layer height, as it derives in a higher value of Young’s modulus. This effect could be related to the fact that porosity is decreased by a lower layer height (and, complementarily, stiffness is increased). Following the same line, the printing velocity proves to increase the stiffness of the specimen as it is lower, probably again by the increase of the overall stiffness.

Of all the tested parameters, both the fill density and the infill pattern had a negligible impact (p-value of the ANOVA test > 5%) and no clear trend, which seems to disagree with the previous analysis. However, it must be considered that the small size of the specimens was derived in a lack of filling, and the geometry was composed basically of boundary layers that have relegated the infill to a second plane in this experimental campaign.

Figure 10B shows that no significant interaction among parameters is observed, as the p-values of them are all greater than 0.05.

#### 3.1.2. Yield Strength

**Figure 11:**
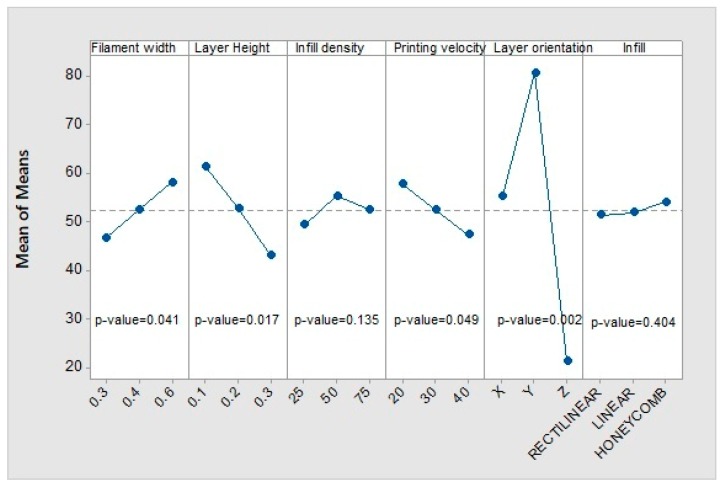
Main effects of means for yield strength.

Figure 11 shows the influence of the printing parameters on the elastic limit. Again, the layer height and the infill pattern do not show a significant influence. The effect of the other parameters on the response follows the same pattern as in the case of Young’s modulus. The most influential parameter again is the printing orientation. With the *Y*-axis orientation, the highest elastic limit is achieved, while the *Z*-axis orientation shows the lowest one. In addition, with the *X*-axis orientation, an intermediate value is achieved with respect to the other printing orientations. The layer height has an influence somewhat higher than that of the filament width, but in the opposite way; as the layer height decreases or the filament width increases, the elastic limit increases. Although the printing velocity has low relevance, a trend is observed: when the velocity decreases, the elastic limit increases.

When analysing the interactions between the different parameters, it is concluded that there is no significant interaction, as the *p*-values in each case are much higher than 0.05. The same happens for the rest of the parameters. This is positive because it means that the influence of the parameters on the response is independent of each other, at least in the ranges of values analysed.

#### 3.1.3. Maximum Strength

**Figure 12:**
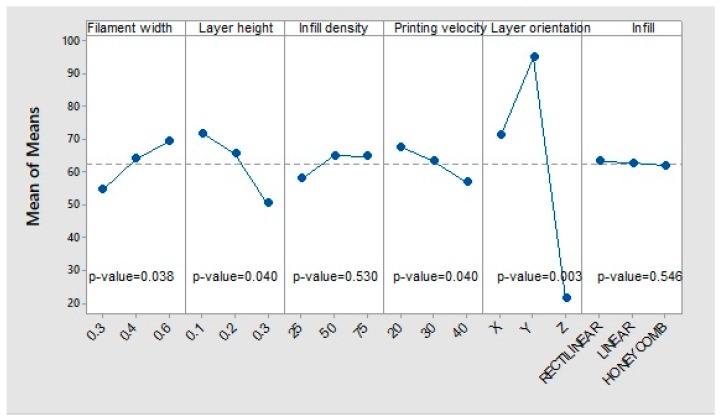
Main effects of means for maximum strength.

The behaviour of the parameters follow the same pattern as the elastic limit case (Figure 12). The layer orientation is still the parameter with the greatest influence on the mean value, followed by the layer height, filament width, and printing velocity, with less influence. Fill density and infill pattern do not have a statistically significant influence.

#### 3.1.4. Maximum Deformation

**Figure 13:**
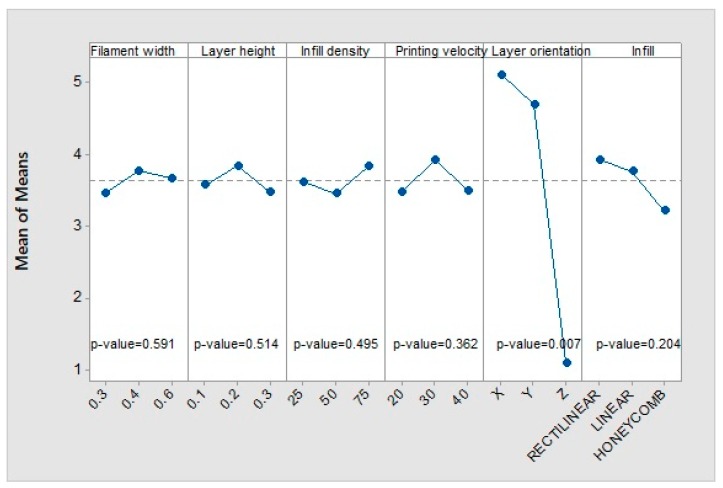
Main effects of means for maximum deformation.

Figure 13 reveals that the only significant parameter is orientation. The *X*-axis and *Y*-axis orientations cause the greatest elongation and the *Z*-axis orientation causes the smallest one. Filament width, layer height, fill density, and printing velocity do not present any pattern or proportionality. The honeycomb fill pattern produces the least effect. Regarding the signal S–N, the only robust parameter is again the orientation.

#### 3.1.5. Summary

In Table 6, a summary of the analysis of the influence of each parameter under study on the different mechanical properties studied can be seen. More green checks indicate that the factor is more influential on the response. Three checks indicates that p-value < 0.01, two checks indicate that 0.01 < p-value < 0.04, one check indicates that 0.04 < p-value < 0.05. The red cross is assigned to the parameters that are not statistically significant (p-value > 0.05). The orientation is the most influential parameter in the zone of both the elastic and plastic behaviour of the pieces tested. The layer height and the filament width are also parameters that influence all of the properties studied, except for the maximum deformation. The same thing happens with printing velocity, but to a lesser extent. In Table 7, the optimum levels of each parameter are shown.

Of all parameters, the lack of influence of infill density deserves a special mention. This observation has already been made by other authors, such as Admed & Susmel (2019) [21] and Andrzejewska et al. (2017) [22]. These authors explain that the mechanical properties of PLA specimens with a 100% infill density depend on three main aspects, namely, the mechanical properties of filaments, the bonding forces between layers, and bonding forces between filaments of the same layer. Decreasing the infill density derives in the loss of bonding strength between filaments of the same layer, regardless of the distance between filaments in the same layer, which is the direct effect of infill density reduction. That is, the effect of changing infill density is more conspicuous when reducing from 100% to any other value, hence the lack of relevance of decreasing it from 75% to 25%. Furthermore, we could add a second fact explaining the lack of influence of infill density on the results, which could be related to the fact that bending specimens are of reduced dimensions, meaning that their mechanical behaviour is governed by their skin and, to a much lesser extent, the infill, which is only comprised by a few layers.

The manufacturing orientation plays a vital role in defining the flexural behaviour of specimens, as stress is normal to the specimen section, and the orientation of the bonding area between filaments shall define the way in which the material processes the stress. This result contrasts with that obtained when the specimens are subjected to fatigue tests, where layer height is the most influential parameter owing to the fact that the limiting factor here is the prevention of crack propagation, and not bearing stress itself [9].

### 3.2. Fractography and Scratch Test

**Figure 14:**
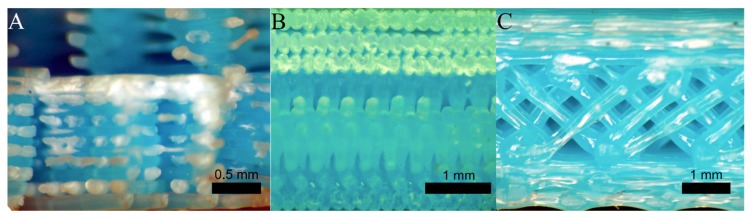
Breaking section, specimens with orientation in the (**A**) X-, (**B**) Y-, and (**C**) Z-direction.

It was already noted that the main factor that determines the strength of the specimens is the orientation of the stacking layers. In Figure 14, the outlooks of the fracture section of some printed specimens in the different directions of the coordinate axes are compared. The specimens printed along the *X*-axis direction (Figure 14A) or along the *Y*-axis direction (Figure 14B) have a slight ductile behaviour with high elongation, good flexural strength, and high rigidity. This reaction is caused by the filaments being aligned with the main stress direction. The resistance depends on the strength of the intra-layer bond and the strength of the filaments. The stiffness and flexural strength are slightly higher in the *Y*-axis orientation specimens. The reason is once again the arrangement of the layers. Although the two orientations have the filaments parallel to the direction of the stresses, the specimens printed in the *X*-axis direction can become delaminated between layers when they are bent. The delamination is produced by the breakage of the weak interlayer bonds. The specimen becomes flexible and is unable to withstand the bending stress, although the intralayer bonds remain intact.

In Figure 14C, the section of the rupture of a test specimen printed in *Z*-axis orientation is shown. These specimens have fragile behaviour and little deformation, low resistance to bending, and low rigidity. This is caused because the layers are oriented perpendicularly to the stresses generated in the specimen during the bending test. For this reason, failure has occurred in the weak interlayer weld without the affection of filament integrity.

**Figure 15:**
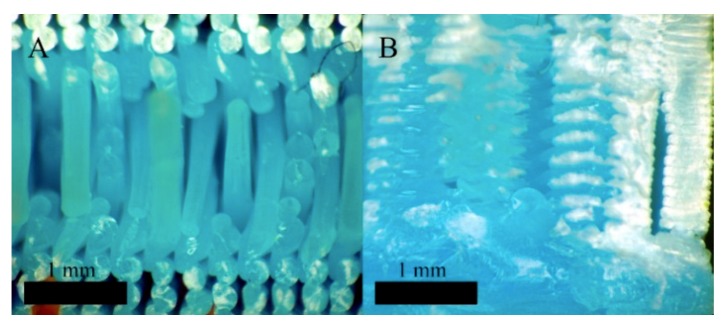
(**A**) Test specimen with a layer height of 0.3 mm and filament width of 0.3 mm. Test specimen 9_2. Microscopic photography. (**B**) Test specimen with a layer height of 0.1 mm and filament width of 0.6 mm. Test specimen 21_3. Microscopic photography.

The second most influential factor is the layer height, followed by the filament width. The smaller the layer height and the larger the filament width, the stiffer and more resistant to bending is the test specimen. This is directly related to the compactness of the threads and the welding between threads (Figure 15).

Figure 15A shows the extreme case with the maximum layer height of 0.3 mm and minimum filament width of 0.3 mm. With these dimensions, the threads are cylindrical and produce low compaction and weak welding owing to the scarce contact surface between threads. On the other hand, as the layer height decreases and the filament width increases, the threads have a flat shape, with a larger welding surface. Figure 15B shows the optimal case with a minimum layer height of 0.1 mm and maximum filament width of 0.6 mm. In summary, the welding surface of the threads, where the micro-welds are produced between the chains of the polymer deposited at the beginning and those of the filament that is then deposited on it, is determinant in the mechanical behaviour. The greater the welding surface, the greater the rigidity and strength of the piece.

**Figure 16:**
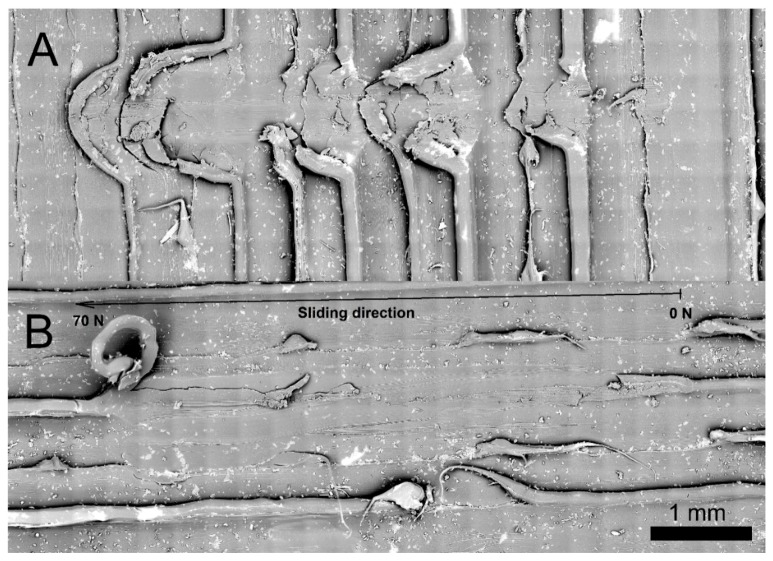
Micro scratch test: (**A**) perpendicular to the printing direction; (**B**) parallel to the printing direction.

Figure 16 shows the micro scratch test tracks in both the (A) perpendicular and (B) parallel direction to the filaments, on the same piece printed in the X-axis direction shown in Figure 14A. It can be seen how, up to the tested force (70 N), the material deforms ductilely without cracking in the base material, as the indenter moves. It also looks like the burrs produced by the extruder are torn. The fact that there are no disclosures between filaments implies that the adhesion between them in the same layer is enough to resist the efforts applied during the test.

**Figure 17:**
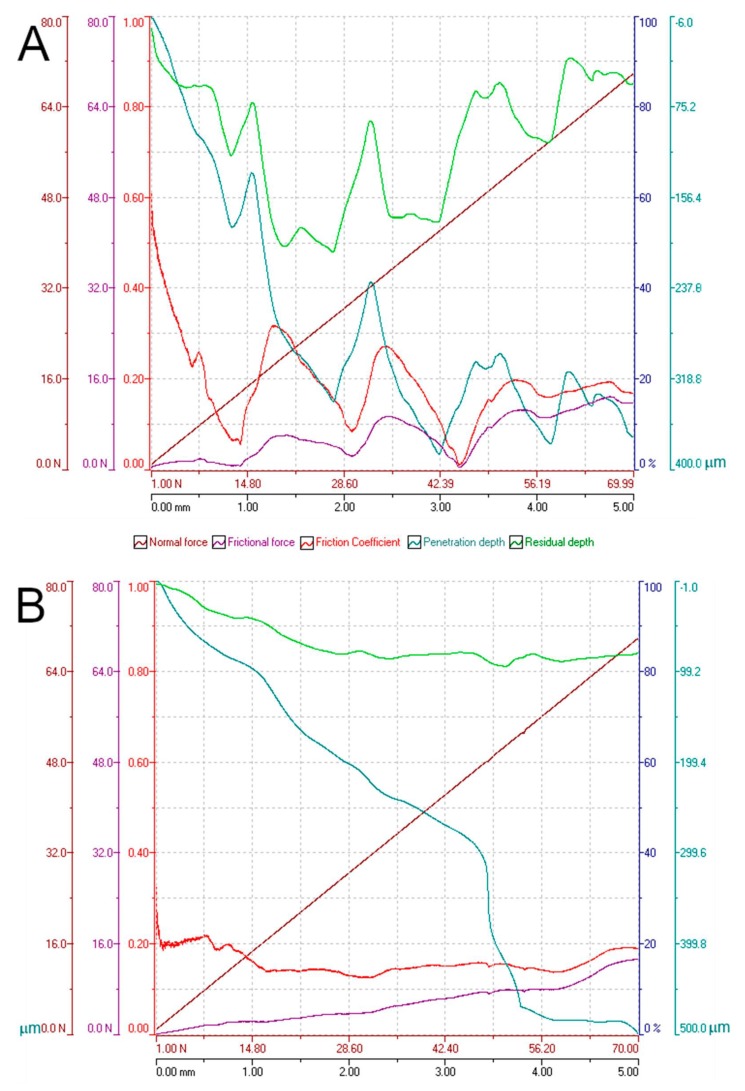
Micro scratch test results: (**A**) perpendicular to the printing direction; (**B**) parallel to the printing direction.

The graph in Figure 17 shows the results of the micro scratch tests: (A) perpendicular and (B) parallel to the direction of the filaments in the range of test forces. The values of normal force, friction force, penetration depth, residual depth, and friction coefficient are clearly observed. While the value of the friction coefficient measured in the perpendicular test shows oscillations, owing to the abrasive wear of the burrs (see arrows in Figure 17A), in the parallel test, its value remains almost constant. It could be possible to sense that these pieces are not showing remarkable wear adhesive.

On the other hand, these burrs left during the extrusion process form channels on the piece surface. If it is true that this worsens the surface roughness of the pieces, they could be useful for retaining lubricant adhered to the sides of the burr ridges; more taking into account that they do not increase their friction coefficient too much, as shown in Figure 17.

### 3.3. Fatigue Test

**Figure 18:**
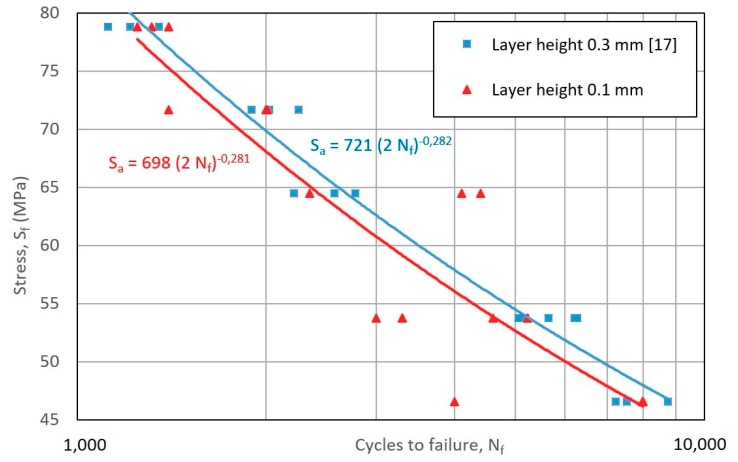
Wöhler curve for the results obtained in this study and those obtained in the work of [14].

The parameter that marks the difference between both curves in Figure 18 is the layer height, being 0.1 mm for the results of this study and 0.3 mm for the referenced study [14].

Although the authors of [14] find that layer height is slightly significant—and although it seems that the following assumption holds: the higher the height layer value, the greater the improvement detected regarding resistance—this cannot be assured, as the errors calculated for the multiplicative factor and the exponent in both equations mean that they can be the same.

Therefore, although a dependence on the layer height is insinuated, the current data do not allow it to assert it.

## 4. Conclusions

The influence of the layer orientation, layer height, filament width, printing velocity, fill density, and infill pattern on the flexural performance of PLA specimens was studied through a Taguchi DOE. The following conclusions can be extracted:

1. The orientation of the stacking of the layers is the most influential parameter in the rigidity, in the flexural resistance, and in the maximum deformation.
2. The layer height and the filament width had a great significance in stiffness and flexural strength and no influence on maximum deformation.
3. Printing velocity had a small, but significant effect on rigidity and flexural strength and no influence on maximum deformation.
4. The fill density and infill pattern had no effect on the studied mechanical properties.
5. The orientation of stacking layers in Y, the layer height of 0.1 mm, the filament width of 0.6 mm and the printing velocity of 20 mm/min was the optimal combination obtained that will allow maximizing rigidity and flexural resistance.
6. The printing direction in *Y*-axis showed the best mechanical behaviour owing to its resistance depending on the strong intralayer bond.
7. The large filament width, the small layer height, and the low printing velocity formed test specimens with better compaction and better welding between wires, and generated a better rigidity and resistance to bending.
8. It could not be ensured that higher layer height improves fatigue life.
9. Depending on the mechanical property to enhance, the combination of optimal parameters to use is different.

## Figures and Tables

**Table 1:** Mechanical properties of polylactic acid (PLA).

Mechanical Property	Value
Yield strength	60 MPa
Elongation at break	6%
Tensile modulus	3600 MPa
Flexural strength	83 MPa
Flexural modulus	3800 MPa

**Table 2:** Parameters and levels used in design of experiments (DOE).

Parameter	Level		
	1	2	3
Filament width (mm)	0.3	0.4	0.6
Layer height (mm)	0.1	0.2	0.3
Fill density (%)	25	50	75
Printing velocity (mm/s)	20	30	40
Layer orientation	X- axis	Y- axis	Z- axis
Infill pattern	Linear	Rectilinear	Honeycomb

**Table 3:** Orthogonal matrix of Taguchi L27 for the DOE.

N°	Filament Width [mm]	Layer Height [mm]	Infill Density (%)	Printing Velocity [mm/s]	LaY-axiser Orientation	Infill
1	0.3	0.1	25	20	X-axis	Rectilinear
2	0.3	0.1	50	30	Y-axis	Linear
3	0.3	0.1	75	40	Z-axis	Honeycomb
4	0.3	0.2	25	30	Y- axis	Honeycomb
5	0.3	0.2	50	40	Z- axis	Rectilinear
6	0.3	0.2	75	20	X- axis	Linear
7	0.3	0.3	25	40	Z- axis	Linear
8	0.3	0.3	50	20	X- axis	Honeycomb
9	0.3	0.3	75	30	Y- axis	Rectilinear
10	0.4	0.1	25	30	Z- axis	Linear
11	0.4	0.1	50	40	X- axis	Honeycomb
12	0.4	0.1	75	20	Y- axis	Rectilinear
13	0.4	0.2	25	40	X- axis	Rectilinear
14	0.4	0.2	50	20	Y- axis	Linear
15	0.4	0.2	75	30	Z- axis	Honeycomb
16	0.4	0.3	25	20	Y- axis	Honeycomb
17	0.4	0.3	50	30	Z- axis	Rectilinear
18	0.4	0.3	75	40	X- axis	Linear
19	0.6	0.1	25	40	Y- axis	Honeycomb
20	0.6	0.1	50	20	Z- axis	Rectilinear
21	0.6	0.1	75	30	X- axis	Linear
22	0.6	0.2	25	20	Z- axis	Linear
23	0.6	0.2	50	30	X- axis	Honeycomb
24	0.6	0.2	75	40	Y- axis	Rectilinear
25	0.6	0.3	25	30	X- axis	Rectilinear
26	0.6	0.3	50	40	Y- axis	Linear
27	0.6	0.3	75	20	Z- axis	Honeycomb

**Table 4:** Test parameters.

Concept	Test Type A	Test Type B
Specimen’s orientation	Z	X and Y
Test speed	1.9 mm/min	19 mm/min
End of test	When break appears in the external fibres	When breaks or deflection = 10.9 mm

**Table 5:** Average results and standard deviations of the material properties. E: Young’s modulus, Rp_0.2_: yield strength, σ_max_: maximum strength, ε: maximum deformation, Std: standard deviation for each property.

#	E (GPa)	Std	Rp_0.2_ (MPa)	Std	σ_max_ (MPa)	Std	ε	Std
1	2.36	0.18	53.8	3.19	64.2	8.18	4.72	1.16
2	3.06	0.07	83.5	0.95	96.0	2.98	4.90	0.64
3	1.79	0.03	11.8	1.74	11.8	1.74	0.70	0.13
4	2.74	0.03	69.7	4.10	79.0	4.97	4.68	1.10
5	1.23	0.10	7.92	1.58	7.96	1.58	0.81	0.24
6	2.71	0.03	60.1	3.09	80.8	2.36	5.85	0.50
7	0.59	0.05	6.71	1.76	6.7	1.76	1.20	0.22
8	2.78	0.11	60.6	3.45	64.1	4.43	3.37	0.32
9	2.81	0.06	65.1	3.61	79.2	6.11	4.91	0.59
10	2.29	0.29	37.1	4.04	37.1	4.04	1.64	0.05
11	3.34	0.19	67.9	3.16	83.7	4.53	4.57	0.17
12	3.69	0.08	95.3	4.26	120.0	1.38	5.34	0.20
13	2.41	0.07	50.2	6.97	72.3	8.23	5.72	0.18
14	3.45	0.33	85.0	3.67	104.6	2.16	4.98	0.17
15	2.07	0.21	26.2	3.34	26.1	3.34	1.49	0.41
16	3.19	0.06	73.4	1.15	83.8	3.87	4.09	0.36
17	1.20	0.09	10.6	1.60	10.6	1.60	1.02	0.13
18	1.44	0.27	26.7	3.25	35.7	4.11	5.09	0.74
19	3.61	0.07	87.4	2.53	95.5	7.35	3.73	0.72
20	3.02	0.27	43.4	3.64	43.5	3.64	1.50	0.12
21	3.23	0.02	70.8	3.51	93.1	4.52	5.09	0.22
22	2.33	0.25	21.3	3.22	21.4	3.22	0.53	0.60
23	2.85	0.19	63.4	6.54	86.4	3.27	5.36	0.64
24	3.70	0.14	90.8	2.28	109.5	4.70	5.11	0.95
25	1.90	0.08	44.0	5.16	60.4	4.43	6.21	0.28
26	2.96	0.15	75.2	3.49	86.7	8.68	4.54	1.56
27	2.30	0.15	25.3	6.61	25.4	6.61	0.91	0.61

**Table 6:** Significance value of the parameters with respect to the answers.

Factor	Elastic Properties		Plastic Properties	
	Young’s Modulus (E)	Yield Strengt (Rp_0.2_)	Maximum Strength (σ_max_)	Maximum Deformation (ε)
Layer orientation	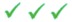	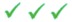	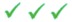	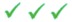
Layer Height	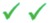	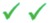	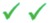	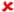
Filament width	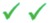	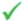	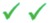	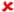
Printing velocity	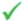	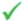	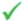	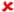
Infill density	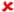	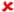	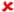	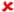
Infill pattern	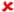	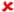	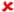	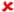

**Table 7:** Parameters’ level to maximize the response.

Factor	Young’s Modulus (E)	Yield Strength (Rp_0.2_)	Maximum Tension (σ_max_)	Maximum Deformation (ε)
Filament width	0.6 mm	0.6 mm	0.6 mm	0.2 mm
Layer Height	0.1 mm	0.1 mm	0.1 mm	0.2 mm
Infill density	75%	75%	75%	75%
Printing Velocity	20 mm/s	20 mm/s	20 mm/s	30 mm/s
Layer Orientation	Y- axis	Y- axis	Y- axis	X- axis
Infill pattern	Honeycomb	Honeycomb	Honeycomb	Rectilinear

## Abbreviations

AM	additive manufacturing
FFF	fused filament fabrication
PLA	polylactic acid
DOE	design of experiments
ANOVA	analysis of variance
